# Reliability of Screening Methods to Diagnose Oral Dryness and Evaluate Saliva Secretion

**DOI:** 10.3390/dj8030102

**Published:** 2020-09-02

**Authors:** Takaharu Goto, Takahiro Kishimoto, Yuki Iwawaki, Keiko Fujimoto, Yuichi Ishida, Megumi Watanabe, Kan Nagao, Tetsuo Ichikawa

**Affiliations:** Department of Prosthodontics & Oral Rehabilitation, Tokushima University Graduate School of Biomedical Sciences, 3-18-15, Kuramoto, Tokushima 770-8504, Japan; c301751010@tokushima-u.ac.jp (T.K.); iwawaki.yuuki.1@tokushima-u.ac.jp (Y.I.); fujimoto.keiko@tokushima-u.ac.jp (K.F.); junchan@tokushima-u.ac.jp (Y.I.); megwat@tokushima-u.ac.jp (M.W.); kan@tokushima-u.ac.jp (K.N.); ichi@tokushima-u.ac.jp (T.I.)

**Keywords:** unstimulated whole saliva volume, stimulated whole saliva volume, oral moisture level, intra-class correlation coefficients, Bland–Altman analysis

## Abstract

In this study, we evaluated the reliability and reproducibility of widely implemented salivary flow rate and oral dryness tests. In experiment 1, twenty young and healthy Japanese participants volunteered to participate. For each participant, the oral moisture (OM) level, unstimulated whole saliva volume (U-WSV), and stimulated whole saliva volume (S-WSV) were measured at the same time on two separate days. In experiment 2, twenty-seven patients who were over 65 years of age volunteered to participate. The OM level and U-WSV were measured at the same time on two separate days. In Experiment 1, the intra-class correlation coefficients (ICCs) corresponding to the S-WSV, U-WSV, and OM level were 0.23, 0.28, and 0.16, respectively, for the young participants. In Experiment 2, the ICCs corresponding to the U-WSV/spitting and OM level were 0.83 and 0.12, respectively, for the older participants. The results of Bland–Altman analysis confirmed the absence of systematic error, with the exception of the OM level results in Experiment 2, which indicated systematic bias. In conclusion, we believe that there is currently no consistent and reliable screening test for assessing salivary flow rate and oral dryness, although the spitting test was determined to be highly reliable.

## 1. Introduction

Xerostomia, the subjective complaint of dry mouth, is a problem and symptom frequently observed in the elderly [1,2,3]. It can occur because of a salivary gland disorder such as Sjögren’s syndrome or systemic disease such as cancer, diabetes, multiple sclerosis, bacterial pneumonia; however, it can also occur as a side effect of post-radiation therapy, multiple drug administration, and in special needs patients [4,5,6,7,8,9,10,11,12,13,14,15]. As the proportion of elderly people continues to increase, so does the number of elderly people who undergo dental examinations and have a chief complaint of dry mouth [16,17,18]. Complaints of dry mouth are also received from patients visiting doctors for preventive health examinations. Xerostomia and decreased salivation have also been reported to be associated with denture incompatibility, dysgeusia, aspiration pneumonia, and difficulties with food intake, swallowing, and speaking [19,20]. Therefore, it is important to clarify the reliability and validity of diagnostic methods for dry mouth and saliva dysfunction.

Various objective methods have been proposed to diagnose dry mouth and salivary dysfunction; some examples include methods to measure the salivary flow rate under stimulated and unstimulated conditions, as well as methods to measure oral mucosal wetness [21,22,23]. However, some of these methods require subjecting the patient to a test that lasts for more than 10 min; this places a heavy burden on the patient and is very troublesome for the operator. Although it is very easy to measure oral moisture (OM) levels using an OM meter, a tool used to identify oral dysfunction, it has been suggested that the detected value does not provide comprehensive information regarding salivary secretion [24]. Most importantly, few studies have evaluated the reliability and reproducibility of the screening test using the test–retest method. Thus, in this study, we evaluated the reliability and reproducibility of widely implemented salivary flow rate and oral dryness tests.

## 2. Materials and Methods

### 2.1. Experiment 1

Twenty young and healthy Japanese participants (9 women and 11 men; mean age, 29.0 ± 5.4 years) from Tokushima University in Japan volunteered to participate in this study. All participants had normal dentition and no stomatognathic or central nervous system complications without medication. The experimental overview and tasks were explained to each participant, and a written informed consent was obtained. Two skilled examiners collected data from each participant over a 2-day period. This study was conducted with the approval of the Ethics Committee of Tokushima University Hospital (approval date: 23 April 2018; No. 3150), and all experiments were carried out in accordance with the approved guidelines.

**Figure 1:**
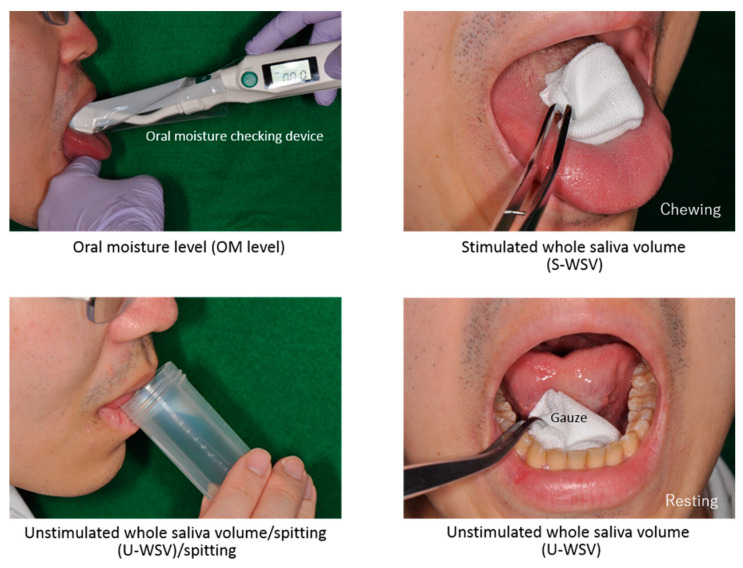
Outline of each measurement test (sialometry).

Figure 1 shows the outline of each measurement test. For each participant, the OM level, unstimulated whole saliva volume (U-WSV), and stimulated whole saliva volume (S-WSV) were measured at the same time under the same conditions on two separate days to ensure test–retest reliability. Furthermore, to maximize measurement accuracy, the measurements were conducted in the following order: OM, U-WSV, and S-WSV.

The Saxon test was employed to measure S-WSV. Each participant was asked to chew a gauze sponge (40 × 40 mm square, dry mass: 2 g) for 2 min; the pre- and post-chewed masses of the gauze sponge were evaluated. U-WSV was determined by measuring and comparing the masses of the gauze sponge before and after being allowed to rest on the hypoglossal area for 2 min. The OM level was measured by positioning an OM checking device (Mucus, Life Co., Ltd., Saitama, Japan) within the central area of the dorsum linguae of the tongue, i.e., 10 mm distal to the apex linguae. This OM checking device utilizes the concept of capacitance to indirectly measure moisture levels. In this study, it was used to measure the OM level of the oral submucosa. The measurement was repeated three times; the mean value was used as the representative value.

### 2.2. Experiment 2

Twenty-seven patients (18 women and 9 men; mean age, 74.7 ± 5.9 years) from Tokushima University Hospital aged above 65 years volunteered to participate in this study. All participants had normal dentition or partial edentulism, with partial dentures that were positioned between the bilateral second molars. The experimental overview and tasks were explained to each participant, and a written informed consent was obtained. Data were collected from each participant over a 2-day period by 11 skilled examiners. This study was conducted with the approval of the Ethics Committee of Tokushima University Hospital (approval date: 26 August 2019; No. 3531), and all experiments were carried out in accordance with the approved guidelines.

The OM level and U-WSV (U-WSV/spitting) were measured at the same time under the same conditions on two separate days within a 1-month interval. The U-WSV/spitting was measured using a conventional spitting method, which entailed asking the participants to spit their saliva into a 25-mL plastic tube, with minimal effort, for a period of 5 min. The OM level was measured by using the OM checking device as described for Experiment 1.

### 2.3. Statistical Analysis

Sex-based comparisons were conducted using the Mann–Whitney U test. Spearman’s correlation coefficient was also determined to clarify the respective relationships between S-WSV, U-WSV, U-WSV/spitting, and OM level measurements. Test–retest reliability was evaluated by calculating the interclass correlation coefficients (ICCs) using the paired values obtained as a result of employing the test–retest method. Bland–Altman analysis was performed to quantify the systematic error in the test–retest paired values [25]. Additionally, SPSS 25.0 (SPSS Inc., Chicago, IL, USA) was used as a platform to perform all statistical analyses, and a significance level of 0.05 was applied.

## 3. Results

Table 1 presents the means of the repeated measurements of Experiments 1 and 2. The mean values of Experiments 1 and 2 were found to be in agreement with those previously reported by researchers who applied the same or similar measurement methods [26,27,28]. Thus, the participants would be considered to be average. Additionally, the differences between females and males were found to be minimal.

Table 2 shows the results of analyzing the correlations between measurements. Spearman’s correlation coefficients for Experiments 1 and 2 were 0.18–0.37 and 0.1, respectively. Additionally, the respective relationships between the results of each type of measurement method were not significant.

Table 3 shows the ICC and systematic error results for Experiments 1 and 2. For Experiment 1, the ICCs corresponding to the S-WSV, U-WSV, and OM level were 0.23, 0.28, and 0.28, respectively, for the young participants. For Experiment 2, the ICCs corresponding to the U-WSV/spitting and OM level were 0.83 and 0.12, respectively, for the older participants. Thus, with the exception of the ICCs corresponding to the U-WSV/spitting measurements, the ICCs tended to be lower. Additionally, the results of Bland–Altman analysis confirmed the absence of systematic error, with the exception of the OM level results in Experiment 2, which indicated systematic bias.

## 4. Discussion

To date, various methods have been proposed to screen for oral dryness and salivary gland dysfunction, which are caused by many different diseases and secondary factors. U-WSV- and S-WSV-based measurement methods, such as the spitting, Saxon, and gum methods [21,22,23], are widely employed to determine the salivary flow rate. Other methods have also been proposed, including the use of a moisture-checking device to measure the impedance of the oral mucosa, filter paper to measure the oral mucosal wetness, or direct clinical inspection of the oral mucosa and saliva [29]. Furthermore, scintigraphy, ptyalography, and lip gland biopsies have also been presented as tests that directly evaluate salivary gland function and yield detailed information [23]. Although a wide variety of methods have been investigated, how the results of oral dryness tests relate to the salivary flow rate has yet to be elucidated; while some studies indicated a significant positive correlation, others reported the lack of a correlation [24].

Generally, the consistency of current measurement methods is low. One reason for this is that during rest, approximately 70% of saliva is produced by the mandibular/sublingual glands, whereas approximately 50% is produced by the parotid gland under stimulatory conditions [26]. Anzai et al. suggested that the OM level does not always accurately reflect the extent of oral dryness because the OM level tends to be low in mouth-breathing patients with a high salivary flow rate [24].

It should be noted that in this study, there was no significant relationship between the S-WSV, U-WSV, and OM level results of Experiments 1 and 2. Additionally, there is no gold standard method for measuring the salivary flow rate and oral dryness.

Regarding the reliability of the tests, the ICC corresponding to the U-WSV/spitting method had the highest value (0.83). Here, we applied the commonly used Landis and Koch guidelines for the ICC: 0.00–0.20 indicates slight agreement, 0.21–0.40 indicates fair agreement, 0.41–0.60 indicates moderate agreement, 0.61–0.80 indicates substantial agreement, and 0.81–1.00 indicates nearly perfect agreement [30]. This means that the U-WSV/spitting method was “nearly perfect,” and that the reliability levels of the other tests were very low. These results were found to be consistent with those of a study by Navazesh et al., in which spitting and draining methods demonstrated to be more reliable and reproducible were performed [31]. However, spitting and draining methods require the ability to spit and drain the saliva out of one’s mouth; thus, it is difficult to apply these methods to nursing care patients and those with cognitive impairment.

As previously mentioned, only the OM level values for the older participants were found to have systematic bias; however, this bias was fixed. This bias may be related to the measuring device itself; however, the origin remains unclear, because the OM level results for the younger participants did not indicate any bias. In the present study, skilled examiners performed all the measurements; thus, bias among examiners was assumed to be low.

The present study has several limitations. First, this study was divided into two experiments, and the historical data were used in Experiment 2. Thus, it has inconsistent experimental design, i.e., different measurement intervals, different tests in two experiments, and insufficient sample size. In terms of the different intervals, there were no changes in participants between the intervals. The use of different tests was intended to present information on more tests. The expected sample size was approximately 30, assuming with α = 0.05, 1 − β = 0.80, and effect size described in a previous report [24]. While the sample size in Experiment 1 was small, the post hoc test using categorized variables suggested that the statistical power of the sample size in Experiment 1 was acceptable. In Experiment 2, we could not conform to the sample size demanded by statistical analysis as historical data was used and the sample number was slightly less than what was statistically required. However, the results from both experiments support and strengthen our argument. Second, the possibility of participant selection bias cannot be denied. So-called normal participants without the complaint of dry mouth were used. In Experiment 2, the older participants were controlled to the underling disease, but the bias by disease was not statistically analyzed. This study concerns just the reliability of each test using the ICCs of test–retest. It has been suggested that the subjective assessment of patients is also an important factor to diagnose dry mouth. Second, In conclusion, we believe that there is currently no consistent and reliable screening test for assessing salivary flow rate and oral dryness, although the spitting test was determined to be highly reliable. Simpler and more universally applicable screening methods should be developed in the future.

## Figures and Tables

**Table 1:** Means and standard deviations of each test.

Variables	Experiment 1 (Young People)	Experiment 2 (Older People)
Age (years)	29.00 ± 5.36	74.70 ± 5.91
Stimulated whole saliva volume Saxon test (g/min)	1.82 ± 1.09	-
Unstimulated whole saliva volume		
Gauze swab (g/min)	0.28 ± 0.43	-
Spitting (mL/min)	-	0.39 ± 0.28
Oral moisture level	28.74 ± 2.05	28.41 ± 1.67 *

* Statistical difference between women and men (*p*-value < 0.05).

**Table 2:** Spearman’s correlation coefficients between mutual tests.

**Experiment 1**			
**Variables**	**S-WSV**	**U-WSV**	**OM Level**
S-WSV	―	0.29 (*p* = 0.21)	0.37 (*p* = 0.11)
U-WSV	―	―	0.18 (*p* = 0.44)
OM level	―	―	―
**Experiment 2**			
**Variables**	**U-WSV/Spitting**		**OM Level**
U-WSV/Spitting	―		0.01 (*p* = 0.97)
OM level	―		―

S-WSV; Stimulated whole saliva volume, U-WSV; Unstimulated whole saliva volume, OM; Oral moisture.

**Table 3:** Intra-class correlation coefficients and systematic biases of each test.

	Intra-Class Correlation Coefficients	Bland-Altman Analysis			
		Fixed Bias		Proportional Bias	
		95% Confidence Interval	*p*-Value	Correlation Coefficient	*p*-Value
**Experiment 1**					
S−WSV	0.23	−0.30~0.97	0.28	0.24	0.32
U−WSV	0.28	−0.02~0.26	0.08	0.28	0.23
OM level	0.16	−1.59~0.75	0.46	0.11	0.96
**Experiment 2**					
U−WSV/Spitting	0.83	−0.11~0.01	0.12	−0.35	0.08
OM level	0.12	0.26~1.84	0.01	−0.28	0.16

S-WSV; Stimulated whole saliva volume, U-WSV; Unstimulated whole saliva volume, OM; Oral moisture.
